# Experiences of soft skills development and assessment by health sciences students and teachers: a qualitative study

**DOI:** 10.1186/s12909-025-07289-2

**Published:** 2025-05-19

**Authors:** Meike van den Beuken, Isis Loos, Esther Maas, Jonáh Stunt, Lothar Kuijper

**Affiliations:** 1 Department of Health Sciences, Vrije Universiteit Amsterdam, Amsterdam, Netherlands; 2 Menzies Institute for Medical Research, University of Tasmania, Hobart, Australia; 3 Public Health en Primary Care, Leids Universitair Medisch Centrum, Leiden, Netherlands

**Keywords:** Soft skills, Development, Assessment, University students

## Abstract

**Background:**

Soft skill development is of growing importance in higher education. However, the definitions of soft skills are unclear and soft skill development and assessment in higher education are underexplored. Therefore, the research question of this study is as follows: What are the experiences of teaching staff and students of the bachelor’s internship in health sciences regarding soft skills development and assessment?

**Methods:**

For this qualitative study, using a phenomenological approach, sixteen participants (ten university teachers and six bachelor students) were interviewed via semi structured interviews, based on the theoretical framework of Nicol & Macfarlane’s model of self-regulation. The results were analysed through thematic analysis.

**Results:**

The experiences of soft skills were divided into eight themes related to development and assessment. In terms of development, it is [1] important to show a growth in soft skills. This development depends on [2] the perceived guidance provided by teachers [3], the type of internships, and [4] the perceived supervision in soft skills. Regarding assessment [5], teachers often struggle to evaluate soft skills, highlighting [6] the need for durable assessment of soft skills. Additionally [7], both students and teachers feel a need for more focus on soft skills in education. Furthermore [8], several prior factors, such as acknowledgement of soft skills and self-reflection skills, are mentioned to optimise student development.

**Conclusion:**

This study highlights the importance of soft skills development and assessment in higher education. A clear definition of soft skills is recommended. Our findings demonstrate how teachers struggle with their role in soft skills supervision and assessment, with the consequence that students experience a lack of supervision and assessment in soft skills development.

**Supplementary Information:**

The online version contains supplementary material available at 10.1186/s12909-025-07289-2.

## Background

Over the last decades, it has become increasingly apparent that soft skills (*“soft skills include personality traits*,* sociability*,* language fluency and personal habits”* [1]) are an important factor for professional success [2, 3]. It has been repeatedly demonstrated that the accompaniment of soft skills with hard skills *(hard skills include specific competencies or technical skills that can be measured and often validated* [4]) benefits many occupational outcomes [1, 5, 6] and it is expected that soft skills will even grow more important in the coming decades. However, soft skill education is only a small part of the curriculum in higher education [7]. Furthermore, the prevention behaviours of COVID-19 had an influence on the social norms and soft skills development of the students who studied during that time [8]. During the pandemic, the rapid development of online education was mostly driven by survival considerations, with a strong focus on hard, rather than soft skills [9]. This resulted in a gap of soft skills development for that cohort [10]. Other important change in working environments are new technologies such as artificial intelligence like Chat GPT, machine learning, cognitive computing, and deep learning. This continued development in the 21st century impacts organisations and individuals because human jobs might be exchanged for computers. Soft skills, e.g. emotional self-awareness, stress tolerance, social and flexibility skills [11], will become more important and will enable people to work together and with artificial intelligence in a complex way, as they include the ability to adapt to the constantly changing world [11]. These developments highlight the need to incorporate soft skills teaching in university programs.

Both hard and soft skills are necessary for professional development and success [4]. Hard skills can be defined as specific competencies or technical skills that can be measured and often validated [4]. Soft skills are generally perceived as less tangible, and therefore the definition of soft skills is more ambiguous. Several authors have provided slightly different definitions and descriptions of soft skills, often using domains to explain the concept. The definition of soft skills used in this study [1] is as follows: *“skills that complement an individual’s hard skills and that can enhance interactions*,* performance and career development. Soft skills include personality traits*,* sociability*,* language fluency and personal habits”.* Hard and soft skills are frequently presented in a binary classification. This might not be appropriate anymore, as they are intertwined and interdependent [4]. Where the current focus in education is more on hard skills, there is an increasing recognition of the importance of soft skill education [12].

The striking focus on hard skills assessments in higher education highlights an underlying assumption that these skills gear university students with the best equipment for their next career step. This is, however, a rather debatable and outdated assumption. In 2019, a set of skills was required to adequately manage complex problems, predicting an increasing demand for soft skills, such as learning ability, collaborative skills and social understanding [13]. These skills specifically pertain to public health and deserve a greater focus in the assessment of university students. This has been widely acknowledged by both students and employers [14] but is also visible in the career skill requirements of external stakeholders [15].

Internships are a great possibility for soft skill development, by partaking in student collaboration, meaningful discussions with peers, and reflection on their professional role as a team member [16, 17]. The Vrije University Amsterdam (VUA) with its identity “Education focuses on students’ personal development” has a Bachelor of Science (BSc) program of Health Sciences. The BSc program’s pinnacle is the bachelor internship, where students perform their own research for three months, supervised by a teacher from the university staff. The BSc-internship results in a scientific report about their research, which is assessed by two examiners. While the teacher assesses both the report and the student’s attitude, a second examiner only assesses the scientific report. As a consequence, the student’s grade leans heavily on the scientific quality of their research. Specifically, the assessment of hard skills (e.g. writing skills, statistical skills) prevails over soft skills (e.g. independence, project ownership, teamwork skills, and motivation)], despite the fact internships are particularly useful for enhancing soft skill development [17].

Currently, it remains unclear if VUA students are aware of their soft skills and their development, and what kind of development they expect to achieve during their bachelor internship. Also, it seems unclear to the teachers how soft skills, or soft skill development, should be guided and ultimately incorporated in the student’s grade. This ambiguity results in an inconsistent approach to soft skills education, where its assessment and integration remain secondary to hard skills. As a consequence, students may not receive the necessary support to develop essential soft skills, potentially leaving them underprepared for the demands of the modern workforce. However, there is limited knowledge on how soft skills should be effectively fostered and assessed within the Dutch context of higher education internships. Therefore, the research question of this study is: What are the experiences of teaching staff and students of the bachelor’s internship in health sciences regarding soft skills development and assessment?

## Methods

### Framework

**Fig. 1:**
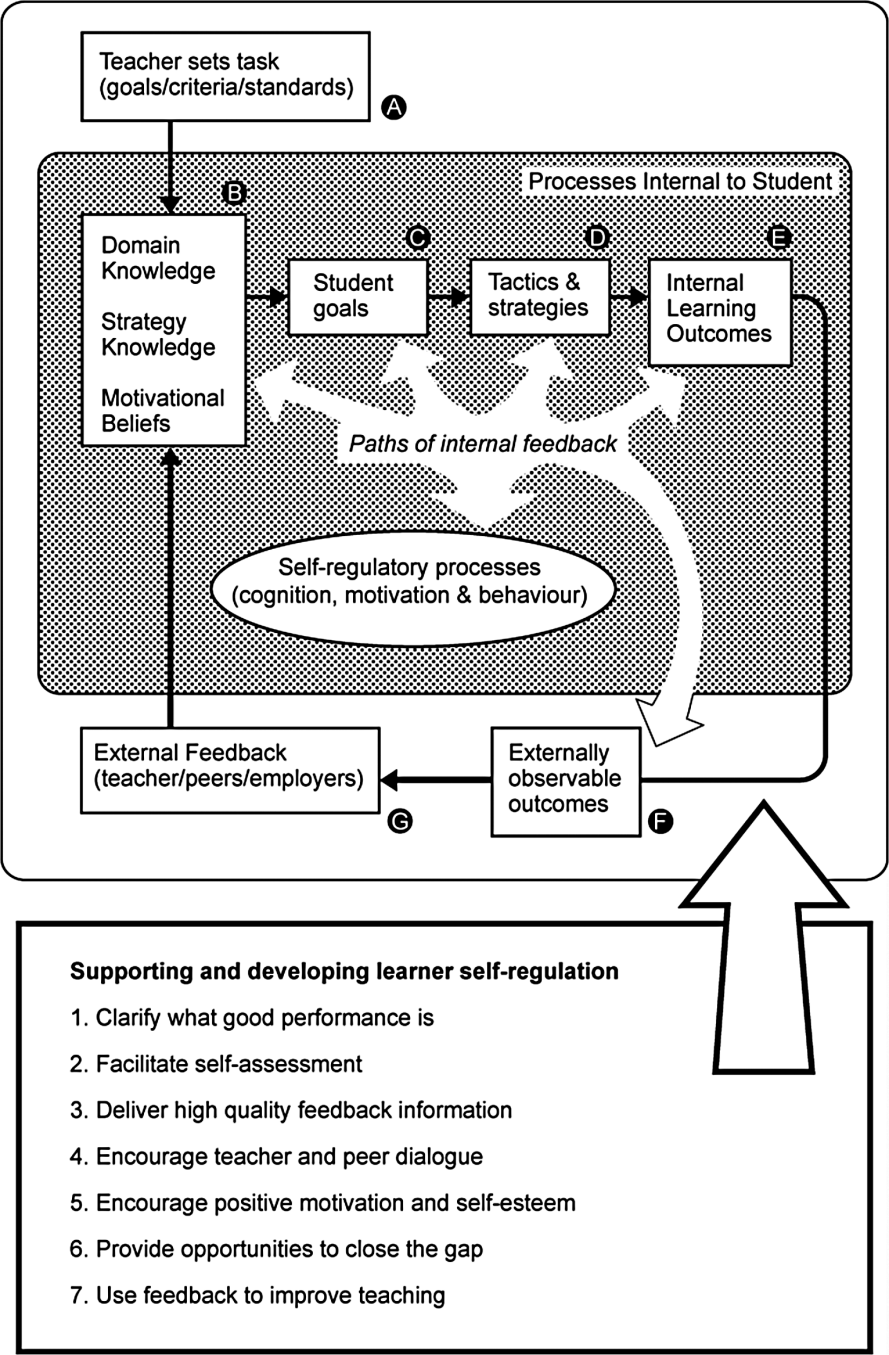
A model of self-regulated learning and the feedback principles that support and develop self-regulation in students

The theoretical framework of this study consists of Nicol & Macfarlane’s model of self-regulation and feedback [18]. This model focuses on self-regulated learning and formative assessments with seven principles of good feedback practice (Fig. 1). The construct of self-regulation refers to the extent to which students can regulate aspects of their thinking, motivation, and behaviour during learning. The development of self-regulated learning in soft skills is a continuous process [19], and this framework helped develop and focus this study. In this study, the self-regulation model was used in different ways. First, it was used as a basis for understanding the concept of self-regulated learning in soft skills. Second, the model was used for data collection and data analysis. The model served as a guideline for setting up the interview guide (see Appendixes 1 and 2).

The framework is suitable for the university setting, especially because the VUA has formulated a new overall vision in 2021 in which education focuses on students’ personal development. Hence, the principle of self-regulated learning aligns with VUA educational visions. One of the core VUA values of the new identity is “The student is primarily responsible for their own study career and own (personal and responsible) study success. Through our education, we stimulate the autonomy and self-management of students [20].”

### Study design

To explore the experiences of soft skills development and assessment, a qualitative descriptive methodology using a phenomenological approach was applied. A phenomenological approach seeks to explore and understand individuals’ lived experiences of a particular phenomenon emphasising the importance of exploring the meaning and essence of those experiences. By focusing on subjective meanings and lived experiences, participants are given a voice, which helps to understand how and why individuals experience a phenomenon in a certain way [21]. The iterative process of data collection and analysis is made effective by thorough exploration and validation of the data.

### Context

The Bachelor program of Health Sciences at the VUA is a three-year program with an interdisciplinary focus on public health and a strong methodological basis. Students are trained to become health scientists with the aim of answering current public health issues from different perspectives and developing creative solutions that improve the health of (international) society.

The program’s last phase is the bachelor internship, where students perform their own research for three months, supervised by a professor from the university staff, who is their teacher. The internships either take place in a group with other students or individually. The students write a scientific research report (5000-10.000 words) within three months, based on scientific literature and qualitative or quantitative data-analyses, to answer a health sciences related research question. They have weekly meetings with their supervisor, and if applicable their group of fellow students. Both group and individual internships have a great potential for developing soft skills. During the internship students have a professional relationship with their teacher and fellow students. The professional interaction and skills that are needed for this provide an excellent opportunity to grow and reflect on their development. The focus on these interactional soft skills is important in learning, reflecting, and assessing, during the bachelor’s program, the internship, and the students’ future career.

The internship is graded on different aspects: 70% is based on their scientific research report, through which mostly hard skills, such as data collection and academic writing, are assessed. The assessment of soft skills is captured in performance (work pace, practical research skills) (20%), attitude (cooperation, processing feedback, motivation, independence, ownership) (pass/fail), and 10% for the oral presentation. The current assessment form can be found in Appendix 3. During the internship there is one other assessment moment: the ‘go/no go moment’. Four weeks after the start of the internship, students are assessed for their introduction and methods and attitude they showed so far. If they get a ‘go’ they can continue their internship, but if they get a ‘no go’ they can get two weeks to show progress, or the internship is terminated.

### Participants

Purposive sampling was used to recruit eligible participants. Purposive sampling is a nonprobability sampling method in which participants are intentionally selected based on characteristics and experiences related to the research question [22]. The participants were selected because they were good representatives of the teachers and students of the health sciences department.

To be included, students had to complete a BSc internship, or teachers had to supervise a BSc internship. Students who had participated in an internship more than one year prior were excluded. There was an additional focus on recruiting external teachers, as the department mostly has internal teachers. All participants in this study were approached through email by the researcher of this study (MvdB). The recruitment continued until the different type of internships (internal, external, group, individual, quantitative, qualitative, both) were represented within the participants and data adequacy was reached, meaning that the data were rich and sufficient to generate meaningful, theoretically informed insights [23].

### Data collection

Individual semi structured interviews were conducted with the aim of obtaining insight into the experiences teachers and students have regarding soft skills development and assessment in the bachelor’s internship. The role of the researcher when using a phenomenological approach involves bracketing biases, deeply engaging with participants, interpreting meanings and reflexively navigating their influence on the study. The researcher is thus aware of its own assumptions, biases and prior knowledge about the phenomenon, and their influence on the study.

The interview guide is subdivided into four topics: *learning tasks (goals/criteria/standards)*,* external feedback*,* domain knowledge and motivation of students*,* and internal feedback paths (self-regulatory process).*

Two different interview guides were created, one for teachers and one for students.

The interview guide for teachers started with the definition of soft skills during the bachelor’s internship. Afterwards, the interviewer showed the current assessment form and gathered knowledge about the learning tasks and the specific goals, criteria, and processes during the supervision of the bachelor’s internship. The second part of the interviews consisted of questions about teachers’ external feedback and preferences regarding the assessment. Finally, there were questions focused on the self-regulatory process of students. The interview guide can be found in Appendix 1.

The interview guide for the students focused on gathering information about the experiences of students who had completed a bachelor’s degree in the past year (2022). The students were asked about the content of their bachelor internship, the definition of soft skills, and learning tasks such as goals, criteria, and standards. The last part of the interviews was focused on supervising during the bachelor’s internship, with a focus on improving soft skills. The interview guide can be found in Appendix 2.

The first two pilot interviews were conducted, one with a qualitative research expert and one with a teacher who focused on soft skill development. After these interviews, minor changes were made in the order of questioning, and the aim was communicated to the participant. The interviews were conducted by the researcher (MvdB) between January and May 2023. The interviews with the teachers lasted approximately 60 min (range 50–70 min). The interviews with the students lasted approximately 35 min (range 30–40 min). The interviews were conducted live on the VUA campus (*n* = 12) or via teams online (*n* = 4) and were voice-recorded. After each interview, the member check was performed verbally. In line with reflexive thematic analysis [24], member checking served to deepen understanding, rather than a measure of verification or validation.

### Data analysis

During the analyses, the self-regulated learning model was used as the basis for the coding process and the code tree (Appendix 4). All interviews were transcribed verbatim. The quotations in the results section were translated from Dutch to English by IL. The interviews were analysed with MAXQDA software. The transcripts were read several times to identify relevant text fragments and analysed by two researchers (MvdB and IL).

Transcripts were coded by two researchers (MvdB and IL). The researchers conducted thematic analysis, a reflexive method for identifying, analysing, and interpreting patterns of meaning (or “themes”) within qualitative data [22]. The process consists of (1) Familiarization: actively engaging with the data to identify meaningful patterns; (2) Generating initial codes: systematically coding relevant data segments; (3) Searching for themes: clustering related codes into potential themes; (4) Reviewing themes: refining themes for coherence and analytical depth; (5) Defining and naming themes: clearly articulating each theme’s essence; and (6) Writing the report: making a compelling, interpretative narrative. Themes do not “emerge” from data, but are actively generated through the researchers’ interpretation, requiring reflexivity, conceptual depth, and a critical, iterative approach to analysis [25].

The two researchers independently performed the initial coding. Subsequently, the findings were discussed until a consensus was reached. Following this, the codes were grouped into categories, which in turn were covered under subthemes belonging to the main themes.

### Ethics

The study protocol was approved by The Research Ethics Review Committee of the Faculty of Science, Vrije Universiteit Amsterdam (BETCHIE), with review number 22–48. All participants were informed in writing and face-to-face about the aims, implications, and ethical aspects of the study. Informed consent was signed by all the participants. In addition, the interviewer indicated in each interview that it was not mandatory to answer if the participant was not comfortable with the question. This was especially important as the interviewer had a prior relationship with all participants. Some of the students were former students and all teachers were colleagues.

## Results

### Participant characteristics

This study included sixteen participants—ten teachers and six students—as shown in Table 1. Among the teachers, nine women and one man were interviewed; the students were all female. This corresponds to the male‒female ratio (10-90%) in teachers and students at the study Health Sciences at the VUA. The teachers had multiple years of experience with bachelor’s internships. Three of the interviewed teachers also had experience as internship coordinators in previous years, in which they arranged all the placement and grading of the internships in those years. The six students who completed the BSc thesis were from the same cohort (March - June 2022).

**Table 1:** Description of the participants– Teachers and students BSc health sciences internship

Characteristics	Teachers *N* = 10	Students *N* = 6
Sex		
• *Female* • *Male*	9 (90%) 1 (10%)	6 (100%) 0 (0%)
Supervising experience in years		
• *0–10 years*	2 (20%)	NA
• *11–20 years*	5 (50%)	
• *Unknown*	3 (30%)	
No. of supervised students over the years		
• *0–25*	1 (10%)	NA
• *25–50*	3 (30%)	
• *> 50*	5 (50%)	
• *Unknown*	1 (10)	
Internship characteristics ^a^		
• *Internal*^*b*^	6 (60%)	4 (66.7%)
• *External*^*c*^	3 (30%)	2 (33.3%)
• *Both*	1 (10%)	0 (0%)
• *Group*^*d*^	4 (40%)	4 (66.7%)
• *Individually*^*e*^	6 (60%)	2 (33.3%)
• *Qualitative*^*f*^	1 (10%)	2 (33.3%)
• *Quantitative*^*g*^	5 (50%)	4 (66.7%)
• *Both*	4 (40%)	0 (0%)

a. Multiple answers were possible

b. Internal internship: internship was performed within the VUA or Amsterdam University Medical Center

c. External internship: internship was performed at an external scientific organisation, supervised by a VUA teacher and an one-site teacher, who has at least a PhD

d. Group internship: a group of students have the same main topic or research project, where the execution of the internship is done with the group, but with an individual research question and thesis

e. Individual internship: student is the only student within that topic with that teacher, executes the internship individually

f. Qualitative internship: Internship with a qualitative data analysis, often using focus groups or interviews.

g. Quantitative internship: Internship with quantitative data analysis, using statistical models

### Themes

**Fig. 2:**
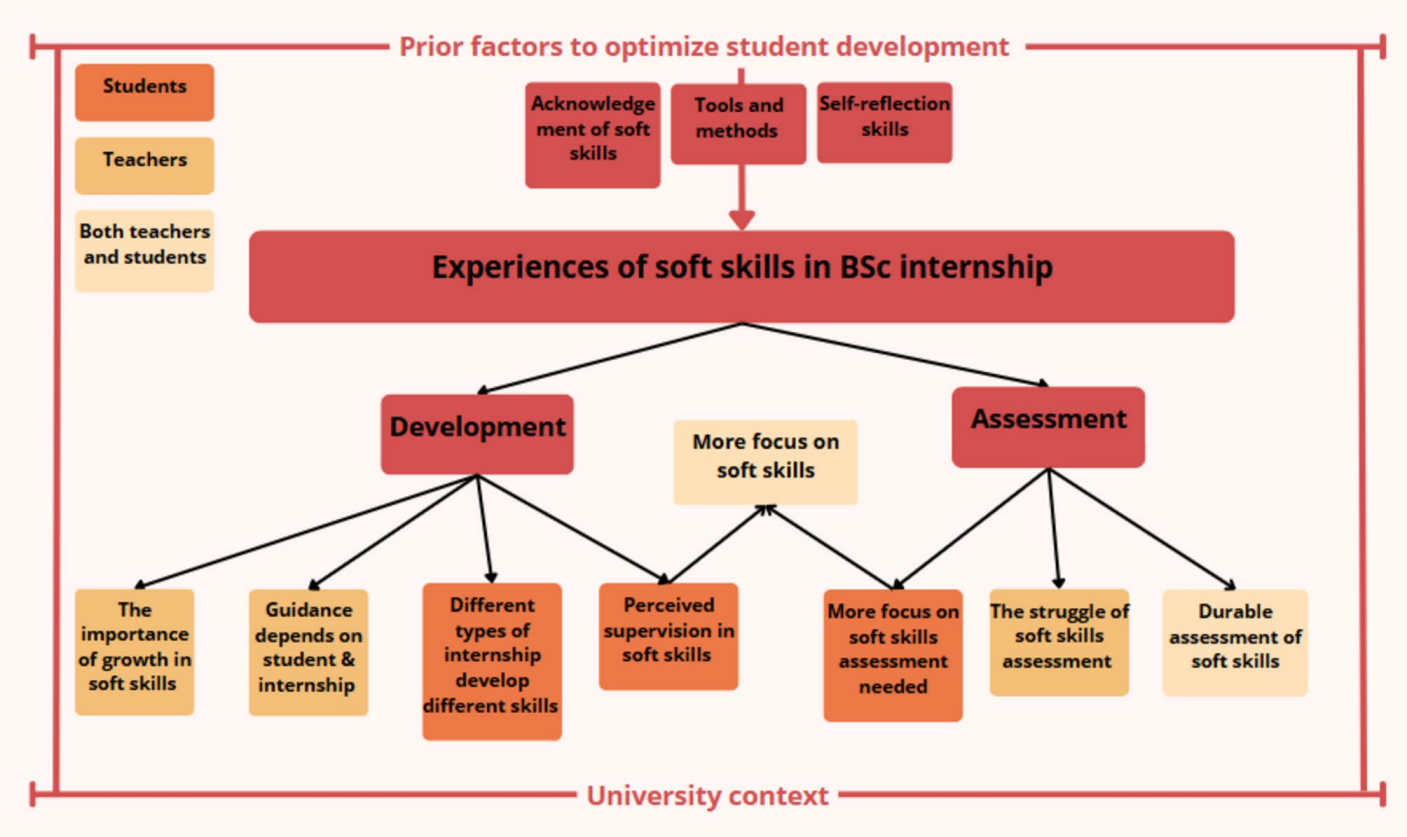
The themes and their underlying relationships

The experiences of soft skills were divided into eight themes for the development and assessment of soft skills (Fig. 2).

In development it is important to show a growth in soft skills. Development is dependent on guidance that depends on the type of student and internship. Different internships have possibilities to develop different soft skills, and development of soft skills is dependent perceived supervision on soft skills.

The assessment of soft skills is often a struggle for teachers, showing a need for a durable assessment of soft skills. Both students and teachers feel a need for more focus on soft skills in education.

Furthermore, several prior factors such as acknowledgement of soft skills and self-reflection skills are mentioned to optimize student development.

‘T’ stands for teachers and ‘S’ for student quotes. The quotations, presented in the results below, are separated by these symbols.

#### Definition of soft skills according to teachers and students

The definition of soft skills in the literature is ambiguous, which was reflected in the participants’ reactions. Teachers understand the given definition of soft skills, but most teachers indicated that they find the definition and meaning of soft skills difficult to describe, especially in the context of the assessment. Some teachers were unsure whether the term soft skills cover the load in this academic context and suggested naming it personal development.

*“And the question is whether to call it soft skills or just personal development…. because soft skills sound a bit like ‘oh I have to do that too’, whereas personal development sounds like opportunities” (T7).*

None of the students knew how to define soft skills.

*“I must honestly say that when I saw your email, I had never heard of the term. So I started googling what does it include and what does it mean. And I think it is mainly about processing feedback, how you deal with it, working together and group work. It said everything on which your attitude is assessed. Well, that is a bit of what I picked out, everything I found on google” (S4).*

When the content of soft skills was explained, students acknowledged the importance of soft skill development during their internship.

*“Yes*,* I think it is good actually because they are important for your later career. It is important to work well together and to communicate well” (S2).*

### Soft skills development in BSc according to teachers

#### The importance of growth in soft skills

Almost all teachers mentioned that it is important that students show a learning curve in soft skills and hard skills. One participant emphasised how we should convey the importance of students’ growth during their BSc program, such as university and supervision, focusing on personal growth. Growth in working independently and independent reasoning are considered particularly important by teachers.

*“However, I also think it is important, especially in these days when we think that everything is very focused on the individual and that everything has to be perfect, we also need to focus on showing that it is okay if you cannot do things in one go and that we kind of need to make students’ growth insightful” (T9).*

Some teachers stressed that soft-skill growth is particularly important during the bachelor’s and internship periods. They indicated that certain knowledge is already assessed in the rest of the BSc program, while the level of soft skills acquisition varies among students. Overall, the teachers agree that, as the BSc internship period is the first time, after two-and-a-half years of studying, for students to perform their own independent research, this is the moment to grow in responsibility and ownership.

*“They have spent two and a half years learning a lot of things. In addition, of course, they applied them, but now they truly have to go through it all… That students learn there is no right or wrong, or not one answer to a problem you mise and make less perfect choices. That’s truly helpful” (T2).*

Teachers consider soft skills to be important for the employability of students. As the BSc internship is the first opportunity for students to perform actual scientific research, this is an excellent period to work on underdeveloped soft skills.

*“I also think they need to grow themselves*,* how do you work together*,* how do you communicate*,* that they make appointments*,* that they keep track of logbooks. So those are more the soft skills you also want to get [in practice]” (R6).*

#### Guidance depends on the type of student and internship

The teachers agreed that their role as a teacher is to provide guidance during the internship. Other important roles are to focus on personal development, to help students achieve their learning goals, and to provide students with tips and tricks during their research process. Regarding their role in soft skills development, some teachers said that they facilitate this personal development process.

“*What are your personal goals? What would you like to achieve*,* what are you already good at*,* and what would you like to work on? And I also explain*,* I would like to support you [student] in it*,* give you [student] feedback on that or we can work on that together” (T9).*

Every teacher sees their role in facilitating this process slightly differently. Every student is different, and there are many types of internships, such as group/individual, qualitative/quantitative, and external/internal. Teachers experience a lack of time for supervising soft skills, and the current focus is on hard skills. The number of contact hours between teachers and students determines the extent to which soft skill development is supervised. The more contact there is, the more supervision of soft skills is present. For some teachers, it remains unclear what their role is in terms of supervising soft skills.

*“On skills, they [students] are hugely variable, so you have to find a connection in where this person is now and what this person needs from me. That is your role as a teacher. That is my vision” (T8).*

In group internships, the focus is mainly on developing soft skills such as cooperation, communication, and intercultural awareness. Teachers indicate that a group internship has different dynamics, where in groups the students first reach out to each other before consulting the teacher. Also, they get more work done in the data collection, as they all collect data with the same purpose and all data is combined to one major dataset. Students individually answer their research question based on the major dataset. This increased the collaboration and communication between students in a group internship. In individual internships, the development of soft skills such as assertiveness, working independently, and ownership is more prominent. In addition, there are different views on the definition and importance of the type of soft skills, as mentioned in the paragraph ‘Definition of soft skills according to teachers and students’. With this in mind, it is important for teachers to first assess the needs of a student.

*“I think they get more out of it [group internships] that they learn from each other…. Giving each other feedback and also cooperation*,* which of course was much more stimulated in groups internship… The final report is all the same just a different focus” (T6).*

### Soft skills development in BSc according to students

#### Different types of internships develop different skills

Students stressed that each form of internship has its own benefits in terms of growing in soft skills. The group internships provide the possibility to discuss the content as well as experiences (e.g., stress) with each other. Students who performed an individual internship stated that it provides the opportunity to work at their own pace and learn to work independently.

*“Well*,* I think you work together more than when you are doing an individual internship. In addition*,* motivation; I think because there are several people there*,* you motivate each other. So that does help you stay up to date” (S5).*

#### Perceived supervision of soft skills

Students indicated that they perceived a lack of supervision in soft skills development. None of the students knew that there was guidance in soft skills or on a personal level. They mentioned that supervision was mostly based on hard skills.

*“I do not want to come across very negative*,* but there was no guidance in soft skills for me. I did not even know that I was supposed to get supervised in soft skills. For me*,* it was more supervising for my thesis*,* so I actually got constant improvements on the work I was doing*,* but not that I had to grow as a person during my thesis as well” (S3).*

After further questioning, some students realised that they received supervision on the soft skills of ‘working together’.

*“Well with collaborative group work they did some work on that*,* but the individual part; how you develop yourself but also more the writing*,* your attitude*,* your academic side. I kind of missed the focus on that. I am not saying they did not do anything about that*,* but there was not truly a focus on that” (S4).*

Students would like to have more feedback moments during supervision. Suggestions included feedback in person with the teacher about the growth in soft skills or within the group and teacher during and after the internship.

### Soft skills assessment in BSc according to teachers

#### The struggle of soft skills assessment

Most of the teachers explained that they lack a clear structure in the way they should assess soft skills and therefore struggle. The reasons for this were a lack of interaction with the students because of limited time with the teacher during the internship and a lack of a clear assessment form and rubrics. Therefore, some teachers feel that they assess by feeling. Others are assessing in combination with the knowledge they have from previous supervision. Some teachers mentioned that to provide good assessment, they need more time to interact with the student.

*“Yes*,* it would be helpful if we are told from the study program what the emphasis is for which soft skills we as a study program consider important and which ones we would like to pay attention to*,* both during the study program and in the internship” (T9).*

#### Durable assessment of soft skills

To develop a good method for assessment, teachers indicated obtaining a soft skills rubric or method for assessing it. A newly developed assessment form with clear definitions and a more constructive grading system with gradations would contribute to showing a student’s growth in soft skills. Teachers had several ideas about methods for assessing soft skills. More than half of the teachers suggested adding a teaching-learning conversation with the student. A few other teachers suggested combining the (self)assessment with a reflection report or with the oral presentation of the students reflecting on their soft skills development. Other participants preferred an assessment with an end conversation rather than a filled-in form. Also, teachers indicated that it is important to involve the students more in the assessment.

*“I do think teachers have eyes for soft skills*,* but it is nowhere kind of explicitly named and evaluated or tested or say there is nowhere explicitly addressed” (T9)*.

Furthermore, several teachers mentioned that they need support from the health science department and staff. Overall, the teachers mention that they would like to hear from students what they wish to develop or learn. This could then be formulated as a student goal at the start of the internship.

*“So what are the main areas of focus that the student would like to pay attention to and may receive feedback on when thinking about soft skills in this internship?” (T3).*

Assessing an internship requires a balance between assessing hard and soft skills. However, the importance of each skill is different for each teacher. A few teachers mentioned that they would prefer to add a grade to their attitude to compensate for a low grades in hard skills. Other teachers would like to further explain their assessment choices for the students. One teacher said that it should first be clear what the goal of the internship is, after which there can be thought about the balance of assessments in soft skills.

#### More focus on soft skill assessment is necessary

To have a durable evaluation of soft skills within a BSc program, more focus should be placed on soft skills in the educational program for both teachers and students. In addition, several teachers mentioned that they need to receive support from the health science department and staff:

*“What the Department of Health Science needs, above all, is a team of teachers who are having fun in this [assessing soft skills]… What I need is that the Department supports this” (T8)*

### Soft skills assessment in the BSc according to students

#### Durable assessment of soft skills

Students indicated being satisfied with the content of the assessment form. However, opinions on the final assessment differ. All the students agreed that the fail/pass end assessment does not reflect the effort they put into it. Students considered a scale or a grade in the assessment form as an option. The final assessment is currently performed by the teacher using with the assessment form, if applicable in discussion with the onsite teacher. Most of the students preferred this way of assessing, and some students suggested peer assessment.

*“Yes*,* I think you should get a different gradation on the assessing form as well*,* because I think failing and passing are two extremes. I do think there is some kind of middle ground*,* so that could be an option” (S6).*

Students would like to bring in their own learning objective. It was suggested to have a personal conversation with their teacher, in combination with an assessment form, a personal reflection report or a joint reflection on their soft skill goal.

*“I would do a conversation in the beginning to see okay*,* what are those soft skills*,* what do you want to do*,* what do you want to grow in? And then you have a conversation at the end where you actually start reflecting; what happened*,* how did you experience it?” (S3)*

#### More focus on soft skill assessment is necessary

Students were open to focusing more on soft skills and their assessments. They point out that being assessed will increase the urgency to focus on it. One student argued that assessment of soft skills during the entire BSc program is better, as three months is too short to grow in soft skills. Some students would like to receive a grade or a scale in the assessment because this would increase their motivation and attention for soft skills development.

*"Yes, if there is also a grade attached to that, yes, it does motivate you to do your best rather than failing or passing" (S5)*.

#### Prior factors to optimise soft skills development according to teachers and students

The participants mentioned the importance of soft skill development in the entire BSc program, not just during the internship. Although soft skills were found to be important to participants, there was limited knowledge about the concept and the learning process. Students would like to learn what soft skills are and why they are important to develop during their bachelor’s degree and for your future career at an early stage. Both teachers and students suggested methods or tools to optimize student soft skill development during the bachelor program, for example, participating in a workshop or reflection with peers.

*“I think a training course could be very informative. Maybe just before the internship or so that a brief explanation is given of what soft skills mean and how important they can be*,* just a brief explanation and training. I think that could already help and give more insight during the internship” (S6).*

To develop soft skills in the internship, teachers indicated that it is important to develop soft skills throughout all years of the BSc program. They emphasised the importance of reflection skills in particular. Some students stated that they would find it difficult to reflect on themselves, whereas others indicated that this is something that you learn along the way during the BSc.

“*They need the ability to reflect in order to see okay*,* this is something I’m good at. This is something I’m not so good at yet*,* and I hope that during the internship we can say right now that I am not so good at this yet. So that we can offer support there precisely because the internship should be a safe environment where you can and may make mistakes*,* so that eventually you can finish with a pass. I think it is especially important that students are also allowed to make mistakes” (T9)*.

## Discussion

### Main findings

This study aimed to obtain in-depth insight into the experiences of teaching staff and students of the bachelor’s Health Sciences regarding soft skills development and assessment in the bachelor’s internship.

The first result this study showed was that among participants, it was challenging to describe the definition of soft skills. A systematic review on soft skills showed that although soft skills have received growing interest in recent years, the definition of soft skills remains unclear: soft skills are often combined with individual competencies, employability skills, or life skills. Often, no definition of soft skills is given, contributing to the ambiguity of the concept. Additionally, there is no consensus with regard to the classification and explanations of the different soft skills [26]. Signs of ambiguity were also present within our study, as participants provided similar explanations for the concepts of ‘ownership’, ‘leadership’, and ‘taking initiative’. Many of the participants emphasised interpersonal skills such as collaboration and communication as soft skills. These parts are very important within soft skills, but not all-encompassing. However, collaboration and communication are important in working together and are closely related to success in a professional environment [27]. The lack of uniformity and consent regarding the concepts of soft skills hinders the training and assessment of these skills [28]. A clear description of soft skills or a conversation about what soft skills entail would be beneficial for the development, such as given in the introduction. In this regard, it is significant to acknowledge the ongoing debate around the terminology around soft skills in the literature. The paper of Murphy et al., [29] discussed the on terminology around soft skills and nontechnical skills, and argue using the term ‘behavioural skills’ since the skills are used in the interaction between people, and it reflect the nature and importance of these competencies better. Whereas Parlamis and Monnot [30] advocate for using the term “CORE” which stands for Competence in Organizational and Relational Effectiveness. They argue that using the word CORE shifts to a more positive depiction of the term soft skills. Other terms in literature which is related to soft skills are social skills [31], and work-intergrade learning [32]. It may help VUA to convert to one of these alternatives term for soft skills to promote their training and assessment.

Teachers stressed the importance of showing growth in soft skills during the internship period but also in the rest of the BSc program. In line with previous studies [2, 3], this study showed that developing soft skills is important. The focus on personal growth is more important in the process of development, than fixed levels of certain soft skills [33]. In addition, focus on growth can lead to increased student motivation [34]. Moreover, teachers agree that they are responsible for guiding the development of, and growth in, soft skills and that this role depends on both the skills that individual students have at the start of the internship and the type of internship (e.g., group internships focus on soft skills other than individual internships). Additionally, teachers experience a lack of time for supervising soft skills, as the current focus is on hard skills. Guidance in the development of soft skills takes time, commitment and involvement of the teacher, in order to help students grow [1, 33]. Personal guidance by teachers helps students develop soft skills and supports individual growth.

On the other hand, students stated that the type of internship largely determines which soft skills are developed and that they perceive a lack of supervision in soft skills. In fact, they were not aware of the fact there was guidance in soft skills at all. The concept of soft skills has to be clear to both teacher and student in order to learn, develop, and assess soft skills within a university setting. Students can be more involved in the learning aims and evaluation of soft skills in the learning process as studies show that this is beneficial for the development. An example was given by the participants such as joint goal setting of assessment discussions to involve students (early) in the learning process.

Regarding the assessment of soft skills, teachers indicated that they find it hard to assess soft skills because of both student- and internship-related factors and lack of structure and instruments for assessment. The soft skills of teachers themselves are also influenced by their ability to adequately supervise soft skill development [35]. Research on how to contribute to soft skills development shows that to improve soft skills in students, it is important to create situations for active learning [36] and that bridging this gap could be done during this internship [37, 38].

Teachers and students agreed that there is a need for a durable assessment of soft skills. Several suggestions were made, e.g., a conversation, a reflection report, peer review of soft skills, or a grade for soft skills on the assessment form. Although some suggestions have been made by both teachers and students regarding the assessment of soft skills, the most appropriate assessment method remains inconclusive. A potential reason for this may be that the variation in internship projects calls for flexibility in assessment criteria, as each internship presents different experiences, thwarting any call for a one size fits all assessment scheme with clearcut instructions and definitions to be used by the assessors. However, the Dutch-Flanders accreditation system requires uniformity of intended learning outcomes for all students which makes some essential flexibility and problematic [7, 32]. In a study by Succi & Wieandt [39], it was reported that the most common way of assessing soft skills is interviewing, but self-assessments, group interactions, role plays, and presentations were also used [12, 40]. Furthermore, the study of Hadiyanto et al. (2017) [41] aimed to develop a student and graduate assessment rubric for soft skills, hard skills, and competitiveness (SHC) for the University of Indonesia. The results showed that the SHC rubric assessment is reliable, valid, and appropriate for assessing students’ soft skills, hard skills, and competitiveness. Whether the SHC assessment would also be suitable for the Dutch university system could be the subject of further study.

Both students and teachers are open to focus more on soft skills and their assessment. It was acknowledged by both teachers and students that it is important to develop soft skills throughout all years of the BSc program to optimise student development The role of universities in training students in soft skills has been emphasised in several studies [1, 35, 42]. Effective classroom strategies for soft-skill training entail group work, discussions, and presentations [41]. From classroom to practice, internships have been shown to positively affect the soft skill learning process [37, 43]. In previous studies, the importance of training and activities to improve soft skills has been demonstrated extensively. Moreover, it has been concluded that university programs need integrated soft skills training programs with ongoing assessment [44]. However, to our knowledge, studies on effective and coherent training programs are still largely lacking at the VUA and other universities. Students and teachers should get training in what soft skills entail, and how to get them on the agenda of their meetings.

Factors to optimise soft skill development throughout the whole BSc program is attention for soft skills, specifically reflection skills. Some students stated that they would find it difficult to reflect on themselves, whereas others indicated that this is something that you learn along the way during the BSc. In our study, the theoretical framework of the Nicol & Macfarlane’s model of self-regulation and feedback was used to understand the concept of self-regulated learning in soft skills. The concept of self-regulated learning aligns with the overall vision of VUA, of which one of the core values is “The student is primarily responsible for their own study career and own study success. Through our education, we stimulate the autonomy and self-management of students [20].” The results of this study show that there is still a way to go to for students to become self-regulated learners and improve their personal development. Such autonomy could be encouraged by providing adequate supervision and guidance regarding soft skills development. Therefore, teachers indicate it is important to explore the needs of the students regarding which soft skills they want to develop, for example in a focus group or workshop. This includes more time and contact moments with student, and can be seen as an iterative process.

### Strengths and limitations of the study

A strength of this study is that it is the first (Dutch) study to qualitatively explore the opinions of teachers in the field of health and students on soft skills development. Furthermore, this study is the first to explore how soft skills are currently assessed and how they could be improved. By conducting semi structured interviews, the researchers were able to obtain rich information to answer the research question. Moreover, the interview topic list was developed in consultation with a qualitative research expert, and before the first interview started, a pilot interview was conducted. After each interview, a member check was performed to reduce the chances of misinterpretation during the interview. Another strength is the use of a theoretical framework [18] to develop the topic list and guide the interviews and analysis, as this provides structure to all elements of the research process. With regard to the participants, the teachers had considerable experience in supervising and assessing students, and the students just finished their BSc, which reduced the chances of recall bias. Finally, the researchers initially coded independently and discussed the codes until a consensus was reached. This finding adds to the credibility of the study.

A limitation of the study is the sampling method. By using the network of the researchers, the sample was probably biased towards participants who are likely to find soft skills interesting and important. An interesting issue was the fact that the participants were acquainted with the interviewer. On the one hand this could potentially have led to socially desirable answers. On the other hand, the prior relationship may have built report and trust before the interview, encouraging deeper sharing. The sampling method may have been a limitation as it resulted in mostly female participants (100% female students). This was as expected based on the male-female ratio for teachers and students within the Health Sciences department and bachelor program. Nevertheless, it would have been beneficial to have a male student perspective, as they might have had a different perspective on soft skills. Furthermore, some participants were not very familiar with the concept of soft skills. As phenomenology relies on rich, detailed data, this may have had implications for the richness of the data as these participants struggled to reflect deeply during the interview. At the same time, the finding that part of the participants was quite new to the concept, was very meaningful and insightful. Lastly, a limitation was the risk that the interviewer impacted the authenticity of the participants answers, as true epoché (bracketing of preconceptions) is difficult to achieve. Practice bracketing by continuous reflexivity, member checking, and debriefing and discussing with the other researchers minimized this risk and made the interviewer aware of personal biases and assumptions.

### Implications for practice

Soft skills development deserves more focus in academy in general [35, 42]. Our results suggest that soft skills education should take place right from the start of the curriculum, so that students and teachers can set clearcut development goals and expectations for capstone projects. The theory behind the framework of Nicol & Macfarlane’s about self-regulation and feedback will be integrated within the education to understand the concept of self-regulated learning in soft skills. In the Health Sciences program at the VU, a skills track including continuous formative teacher and peer-to-peer feedback will be designed along these lines.

As soft skills are essential for employability and job performance [26], it is important to create an environment where students can safely develop soft skills [45]. To provide a safe learning environment, within the classroom or individual conversations with the teacher, there is a need for ground rules made by students [45]. Furthermore, the use of appropriate language, offensive discourse (e.g. no discrimination or pejorative), and respect towards other students rights’ to hold their positions [46].

The results of the current study have shown that the assessment form (Appendix 3) was not clear enough. Teachers would like to have clear soft skills definitions and a more constructive grading system with gradations which would contribute to showing a student’s growth in soft skills. Although the extent to which any multi-criterion assessment form is appropriate for the intricate process of soft skills assessment is limited [47], the context of the Dutch academic system as well as VUA’s has made us rebuild the assessment form with clearer rubrics (see Appendix 5).For example, the most important soft skills mentioned by the participants are incorporated with a clear definition based on discussion with teachers and students from the department. For a more constructive grading system with gradations to stimulate growth; the words insufficient-sufficient-good-excellent changed to beginning-under development-advanced-expert. To involve students in the assessment from the beginning of the internship, students will have the opportunity to formulate a soft skill goal at the start of the internship to help develop soft skills and to self-regulate learning. The framework used in this study could be applied in education to help self-regulated learning and improve soft skills during the BSc internship.

Acquiring both hard and soft skills, as opposed to knowledge, is increasingly perceived as important, but in this study, there is a focus on soft skills. These concepts of hard and soft skills are highly intertwined, and personal, educational and professional success depends on the capabilities of both of these skills [4]. Therefore, in practice, a more holistic approach with both these skills would be beneficial for students as this reflects the real-world professional environment.

### Recommendations for further research

In the context of our bachelor’s programme, future research should focus on the development and evaluation of the newly developed procedures and changes in education related to students’ soft skills development and assessment at the VUA. This could be done by evaluating the new rubrics and assessment form and investigating the effects of students formulating soft skills goals at the start of the internship to develop soft skills and to self-regulate learning. In a broader context, further research on developing and accessing soft skills should be conducted at different universities and educational programs, especially also in programs where the student and teacher population is more diverse, as this could give more insights. This research could be extended to employers’ perceptions in terms of soft skills for health sciences-related employers.

Soft skills education is gaining momentum, but so is online education. Although there have been successful attempts to develop effective active online tools that help individuals develop soft skills [48], online education can also be a challenge for soft skills development, reducing peer-to-peer contact and replacing mentorship with personalised AI-supported feedback. During the COVID-19 pandemic, the necessity of introducing online classes has exposed the importance of face-to-face education [49]. Soft skills development largely relies on personal feedback and peer-to-peer contact, which suggests that university programs should balance online tools for substance against offline education for transferable and soft skills as an integral part of a bachelor’s program. This is supported by our findings, particularly themes ‘acknowledgement of soft skills and self-reflection’ and ‘the need for more focus on soft skills in education’.

## Conclusion

This study highlights the importance of soft skills development in higher education. Our findings demonstrate that there is currently a lack of clarity regarding soft skills development and assessment and that teachers struggle with their role when supervising and assessing soft skills, with students experiencing a lack of supervision and assessment as a consequence. A greater focus on soft skills and soft skills assessment is considered necessary. For adequate supervision and assessment of soft skills, it is essential that the definition of soft skills is clear for both teachers and students. Additionally, both students and teachers feel that a durable assessment of soft skills is needed. Last, the university should have a clear vision on soft skills development, supervision and assessment that needs to be expressed throughout the BSc program, as this is pivotal for improving soft skills in students.

## Electronic supplementary material

Below is the link to the electronic supplementary material.

Supplementary Material 1: Appendix 1

Supplementary Material 2: Appendix 2

Supplementary Material 3: Appendix 3

Supplementary Material 4: Appendix 4

Supplementary Material 5: Appendix 5

## Data Availability

The datasets generated and/or analyzed during the current study are not publicly available due to the conditions stated by the Ethics Committee to protect the identity of the participant institutions but are available from the corresponding author on reasonable request after being anonymized. Requests to access the data should be addressed to e.t.maas@vu.nl. Anonymised individual participant data (including data dictionary) will be available on request, to researchers who provide a methodologically sound scientific proposal that has been approved by an ethics committee.

## Abbreviations

BSc: Bachelor of Science
S: Student
T: Teachers
VUA: Vrije Universiteit Amsterdam
