# Supplementary material for: Experiences of soft skills development and assessment by health sciences students and teachers: a qualitative study
Source: BMC Med Educ. 2025 May 19;25:724. doi: 10.1186/s12909-025-07289-2 (PMC12087106; doi:10.1186/s12909-025-07289-2)
Supplement: Supplementary file 1 — Supplementary Material 1: Appendix 1 [file 12909_2025_7289_MOESM1_ESM.docx]

**Appendix 1. Interview guide for the teachers**
**Topic list**
Thank you for participating in this interview. Let me briefly introduce myself; my name is Meike, I am working as a junior lecturer at the Bachelor of Health Sciences. As I briefly told you in the mail, with a project group we are looking at soft skill development. By soft skills we mean trainable skills, personal characteristics, skills to do with working with others such as communication with others, but also coping with stress and being able to plan well. How people function by themselves and with others. The aim of this interview is to gain more insight into the supervision and assessment of soft skills in bachelor (group) internships.

The interview lasts about an hour. I would like another verbal permission to record the interview.....

The recording and data will remain anonymous. I think it is important to tell you that should you ever be uncomfortable with a question, or don't want to answer it, please let me know. In addition, you can stop the interview whenever you want. Do you have any questions before we start?
 **Opening:**

How long have you been working as a teacher?

What is your experience in supervising internships?

How many internships have you supervised? What type of internships? group/individual/qualitative/quantitative)

**Learning tasks (goals/criteria/standards)**

Bachelor internships

- What is the function of an undergraduate internship according to the interviewee?
- What should a student have achieved in an undergraduate internship? What is your view on this?

Introducing soft skills. Give definition of soft skills

- In current undergraduate internships (assessment); the focus is on hard skills and thus the report. If there is more focus on soft skills, there will be less focus on hard skills. How big should the soft skills component be? How would you shape this balance yourself?
  - Which soft skills do you think are important?
  - Which soft skills do you want to assess?
- How do you handle the assessment of soft skills during an undergraduate internship?
  - How do you feel about assessment? (Difficult/easy/obvious) Explain what I am referring to; how do they experience the assessment, is this an easy process/difficult process? What do you encounter while assessing?
  - How do you ensure 'reliable' assessment? Or that it goes well?

Review assessment form what is there now

- Are there soft skills that you assess a student on without it being in the form?
  - What is missing from the assessment?

*Explanation: attitude is now pass/fail.*

- How would you like to assess soft skills (e.g. with a grade/? For inspiration Pass/fail grade. Go back to assessing hard skills. What form should the criteria take? Summative/ formative/ satisfactory/good/excellent/ failed/passed.

**External feedback (teacher/peers)**

We have now talked about the current design of the internships; and the role of soft skills in the internships, the assessment and what this might look like in the future; now we would like to move on to a new topic; the supervision of internships.

- How do you see your role as a teacher in relation to soft skills? How do you supervise this?
- What tools do you need to be able to give feedback/guidance on soft skills development?

There are also students working in groups; also defining group placements; different sizes, internal and external; same subject or different subject but with the same teacher. Not just content. 🡪 possibly omit

- How do you envisage a group internship? What is the purpose of a group internship?
- How does it differ from an individual internship?
- How does the group interact with a group internship?

**Domain knowledge and motivation of students**

- What does a student need (knowledge/motivation) in order to develop soft skills? For inspiration: Knowledge or education needs, reflection form.
  - What do you expect from students? How do you gauge the initial situation?

**Internal feedback paths (self-regulatory process) 🡪 possibly omit.**

- Do students (together) engage in conversation about the development of soft skills? Can students reflect on this development? *Safety in placement environment; can students give feedback? Do students enter the conversation by themselves?*
- To what extent can students indicate goals/tasks themselves?
- As a teacher, can you supervise students' self-reflection (self-regulatory process)? How can you do this?

Closing:

Briefly summarize and then ask if the respondent would like to say or add anything else. This is also a great time for yourself to revisit something, if you didn't find a natural moment to do so earlier.

And afterwards:

- Thank the respondent.
- Repeat that it will be anonymized.
- Ask if a member check would be OK.
- Indicate how respondent can reach you if necessary.
- Briefly explain the further process of the survey
