# Supplementary material for: Experiences of soft skills development and assessment by health sciences students and teachers: a qualitative study
Source: BMC Med Educ. 2025 May 19;25:724. doi: 10.1186/s12909-025-07289-2 (PMC12087106; doi:10.1186/s12909-025-07289-2)
Supplement: Supplementary file 2 — Supplementary Material 2: Appendix 2 [file 12909_2025_7289_MOESM2_ESM.docx]

**Appendix 2. Interview guide for students.**

**Topic list**

Thank you for participating in this interview. Let me briefly introduce myself; I'm Meike, working as a junior lecturer at the Bachelor of Health Sciences. As said briefly in the mail, we are working with a project group to look at soft skill development during the final internship of the bachelor's degree in health sciences. By soft skills we mean personal trainable skills, which have to do with functioning well and cooperating with others such as communication, but also coping with stress and being able to plan well. It is all about how people function by themselves and with others. The purpose of this interview is to gain more insight into the supervision and assessment of soft skills in undergraduate (group) internships.

The conversation lasts about half an hour. I would like another verbal permission to record it. The recording and data will remain anonymous. I think it's important to tell you that should you ever be uncomfortable with a question, or don't want to answer it, please let me know. In addition, you can stop the interview whenever you want. Do you have any questions before we start?

**Opening:**

What kind of internship did you do (group/individual/qualitative/quantitative)?

What was your internship about?

What did you like about your internship?

**Introducing soft skills:**

- Do you know what soft skills are? How do you feel about soft skills being assessed in the bachelor?
- In the current undergraduate internships (assessment), the focus is on hard skills and thus the report. If there is more focus on soft skills, there will be less focus on hard skills. How big should the soft skills component be? How would you shape this balance yourself?
  - Which soft skills do you think are important?

**Guidance on soft skill development from internships**

- How did you experience the supervision of soft skill development?
  - How did it go?
  - What did your VU teacher do to encourage your development?
  - Difference between teacher VU and/or external?
- What did you need during your internship to develop soft skills?
  - What would you like to see in terms of supervision (intervision/extra training/earlier teaching/more appointments with teacher)?
  - What do you think the ideal supervision would look like?

**Learning tasks (goals/criteria/standards)**

Review assessment form what is there now

- What do you think of the soft skills assessed in the form?
  - Are there any soft skills that you have developed that are not currently listed?
  - Are there soft skills that are in there, but you don't think are appropriate for an internship?

*Explanation: attitude now passed/failed.*

- How would you assess soft skills (e.g. with a grade/? For inspiration Pass/fail grade. Go back to assessing hard skills. What form should the criteria take? Summative/ formative/ satisfactory/good/excellent/ failed/passed.
  - What to assess - which soft skills?
  - Who assess - External, self, fellow students,
  - How to assess
    - Conversation. self-reflection, report, presentation
    - Grade, failed, passed.
- On what do you think that assessment on soft skills is based?
  - In what way were you able to provide evidence for elements you were assessed on?
- In what way would you like to see that? (report/discussion/personal learning objective/reflection)
- Can you state your own learning objectives for your soft skill development?
  - Could you?
  - Do you need help with that?
  - How would you like to see that?

There are also students working in groups; also defining group placements; different size, internal and external; same subject or different subject but with the same teacher. Not just content. 🡪 possibly omit

- What did you think of a group internship?
- What did you like? What worked less well?

**Closing:**

Briefly summarize and then ask if the respondent would like to say or add anything else. This is also a good time for yourself to come back to something, if you didn't find a natural moment to do so earlier.

And after that:

- Thank respondent.
- Reiterate that it will be anonymized.
- Ask if member check would be OK.
- Indicate how respondent can reach you if necessary.
- Briefly outline the further process of the survey

Aim: to get students' opinions on the system of supervising and assessing soft skills in undergraduate internship. So interview students who did internships last year. Should this fail, then focus more on students who are in their final phase of the undergraduate internship. A total of around 5 students to interview.

1. Posting announcement on Canvas
2. Email students personally.

Include criteria.

Must have completed internship, maximum one year ago, it does not matter where or how they completed internship. Preferably 2022 due to corona signals.
