# Supplementary material for: Experiences of soft skills development and assessment by health sciences students and teachers: a qualitative study
Source: BMC Med Educ. 2025 May 19;25:724. doi: 10.1186/s12909-025-07289-2 (PMC12087106; doi:10.1186/s12909-025-07289-2)
Supplement: Supplementary file 3 — Supplementary Material 3: Appendix 3 [file 12909_2025_7289_MOESM3_ESM.docx]

**Appendix 3. Current assessment form**

| **Assessment Criteria** | |
| --- | --- |
| **Fall** | **Pass** |
| **Collaborating and handling feedback** | |
| The student prefers to go his/her own way and is insufficiently able to cooperate. The student listens to advice but follows it very selectively. The student shows little insight into his/her weaknesses and seems unable to change his/her research/behaviour based on feedback. | The student cooperates sufficiently and is easily included in a group. The student takes advice, feedback and criticism and is able to develop as a result. The student helps others when needed. |
| **Motivation** | |
| The student conducts research because it is required but shows little or no interest. The student is frequently absent and/or engaged in matters not relevant to the research. The student is easily distracted from research tasks. | The student is interested in scientific research and carries out the research neatly, as agreed beforehand. The student spends sufficient time on the research. |
| **Independence and responsibility** | |
| The student does not function adequately without strict guidance from the teacher. The student feels little to no responsibility for his/her activities. | The student mostly works independently, and feels responsible for his/her activities. |
| **Initiative** | |
| The student is expectant, does not participate sufficiently in decision making. The student takes little to no initiative and is reluctant when changes need to take place. | The student takes sufficient initiative and contributes ideas and possible solutions. The student makes decisions (in consultation with teachers). |

| **Assessment criteria implementation** | | | |
| --- | --- | --- | --- |
| **Insufficient** | **Sufficient** | **Good** | **Excellent** |
| **Work pace and planning** | | | |
| The student is unable to stick to the agreed schedule. Cannot adapt the schedule to new circumstances and runs into problems. | The student adheres to the agreed schedule. Asks for help in time if the planning needs to be changed | The student adheres to the agreed schedule. Asks for help in time if the planning needs to be changed | The student adheres to the schedule, adjusting it if necessary and still staying within the agreed time. Student can plan and carry out different activities in parallel |
| **Practical research skills** *Data collection and/or data analysis, working accurately, if applicable: keeping lab journal/ logbook* | | | |
| The student collects required data but works inaccurately or can poorly justify his/her methods or does not know how to complete the data collection. The organisation of data collection is messy and the lab journal/log is not neatly maintained (if applicable). The student has difficulty applying the chosen analysis techniques | Under the guidance of the teacher, the student collects the required data accurately and correctly. If applicable: the lab journal/log has been adequately maintained. The method and analysis techniques have been covered in previous teaching (e.g.: chi-square test, ANOVA, regression analysis). The student can correctly apply the most appropriate analysis technique with some guidance from the teacher. | The student collects the required data independently and accurately. If applicable: the lab journal/log is kept neat and orderly. The student is able to justify the method and procedure followed. The student is able to independently apply appropriate analysis techniques covered in previous teaching (e.g.: chi-square test, ANOVA, regression analysis) | The student collects required data independently and accurately. If applicable: the lab journal/log is excellently kept, in such a way that another researcher can reproduce the research/experiment without any further explanation. The student is able to justify the method and procedure followed. The student learns new skills quickly and needs little guidance. The student selects the most appropriate method of analysis and applies it independently. The student is able to apply more complex techniques with assistance. |
