# Supplementary figures and images for: Experiences of soft skills development and assessment by health sciences students and teachers: a qualitative study
Source: BMC Med Educ. 2025 May 19;25:724. doi: 10.1186/s12909-025-07289-2 (PMC12087106; doi:10.1186/s12909-025-07289-2)

**Appendix 4. Code tree**


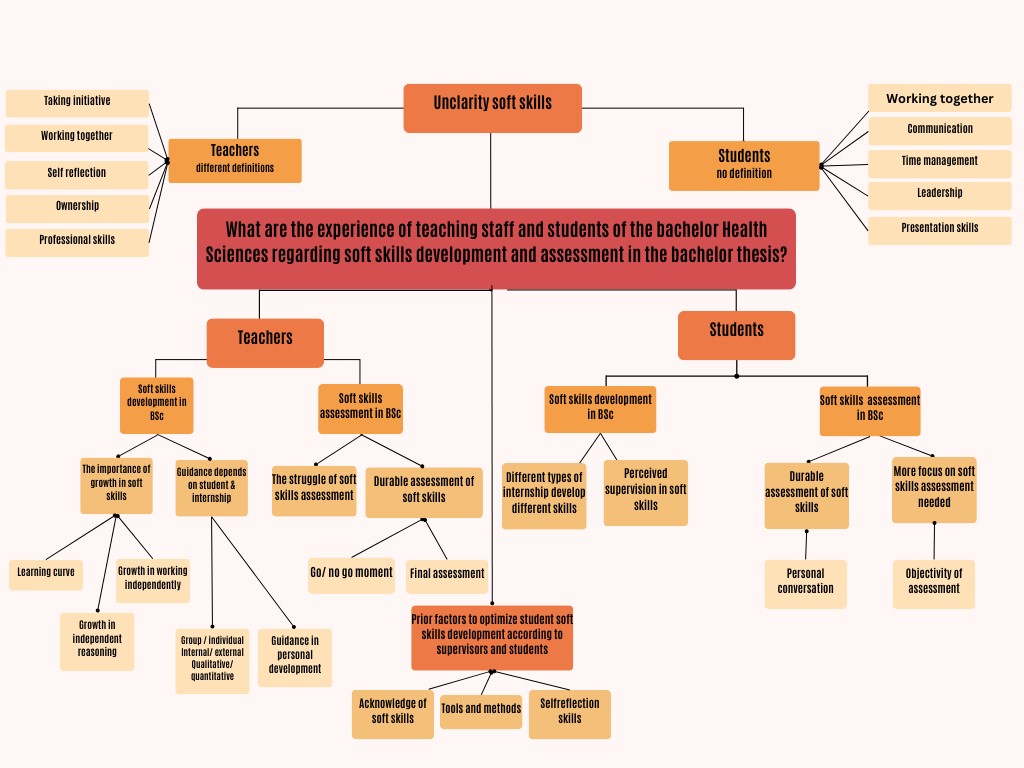

Supplement: Supplementary file 4 — Supplementary Material 4: Appendix 4 [file 12909_2025_7289_MOESM4_ESM.docx]
