# Supplementary material for: Experiences of soft skills development and assessment by health sciences students and teachers: a qualitative study
Source: BMC Med Educ. 2025 May 19;25:724. doi: 10.1186/s12909-025-07289-2 (PMC12087106; doi:10.1186/s12909-025-07289-2)
Supplement: Supplementary file 5 — Supplementary Material 5: Appendix 5 [file 12909_2025_7289_MOESM5_ESM.docx]

**Appendix 5. New developed assessment form**

| **Practical and personal skills** Assessment VU teacher in consultation with daily supervisor  Items can be assessed with the following levels: beginning/under development/advanced/expert | | | |
| --- | --- | --- | --- |
| **Beginning** | **Under development** | **Advanced** | **Expert** |
| **Collaborating** | | | |
| Student is insufficiently able to cooperate. Student communicates poorly or hardly at all. | Student can cooperate in assignment. Student does not communicate constructively or adequately | Student can cooperate and communicates with respect. | Student cooperates excellently and is sufficiently included in a group. Student helps others when needed. Student communicates with respect and contributes to shared understanding. |
| **Motivation** | | | |
| Student conducts research because required, without own interest. Student is frequently absent. | The student is present but shows little to no interest in the study. | Student conducts research as expected of such. Is always present and asks questions. | Student is interested in scientific research and conducts research neatly. |
| **Independence and responsibility** | | | |
| Student does not function adequately without strict guidance from instructor. Student does not feel responsibility for research tasks | The student works independently but feels little responsibility for the outcome. | The student works mostly independently and bears responsibility for the outcome | The student works independently, and feels responsible for his/her activities |
| **Initiative** | | | |
| Student takes little to no initiative in contact moments and is unprepared | Student hardly takes initiative in contact moments. Prepare few questions. | Student takes initiative in contact moments, and prepares well. | Student takes initiative in contact moments, and prepares well. Has own ideas for the research and possible analyses |
| **Coping with feedback** | | | |
| Student is not open to feedback. Follows advice selectively. | Student is not very open to feedback. | Student is mostly open to feedback. | Student takes advice and feedback well and can develop as a result. |
| **Reflection** | | | |
| Student does not ask (critical) questions or participate in discussions.  Student cannot reflect on own choices or development. | Student participates in discussions when encouraged and is able to voice his/her opinion.  Student should be directed to think about personal development. | The student actively participates in discussions and is able to express his/her opinion.  Student actively participates in discussions and is able to put forward his/her opinion. Student is able to reflect on own development process. | The student actively participates in discussions and is able to justify and sustain his/her opinions. Independently asks critical questions regarding content and methodology. Students can reflect on their own development process and suggest points of improvement. |
| **Worktempo and planning** | | | |
| Student is unable to stick to the agreed schedule. Unable to adjust schedule to new circumstances. | The student has difficulty sticking to the agreed-upon schedule and asking for help in time if it needs to be changed. | Student adheres to the agreed-upon schedule. Asks for help in time if the schedule needs to be changed | Student adheres to the schedule, making adjustments as needed and then staying within the agreed upon time. Student can plan and perform different work in parallel |
| **Practical research skills**  *Data collection and/or data analysis, working accurately* | | | |
| The student collects required data, but works inaccurately or can poorly justify his/her methods or fails to complete the data collection. The organization of data collection is messy and the lab journal/log is not neatly maintained (if applicable). The student has difficulty applying the chosen analytical techniques. | The student, under the guidance of the supervisor, collects required data accurately and correctly. If applicable: lab journal/log has been adequately maintained. Method and analysis techniques have been covered in previous instruction (e.g., chi-square test, ANOVA, regression analysis). The student can correctly apply the most appropriate analysis technique with some guidance from the supervisor. | Student collects required data independently and accurately. If applicable, the lab journal/log is neatly and clearly maintained. The student is able to justify the method and procedure followed. The student is able to independently apply appropriate analysis techniques covered in previous instruction (e.g., chi-square test, ANOVA, regression analysis). | The student collects required data independently and accurately. If applicable: the lab journal/log is excellently maintained, in such a way that another researcher can reproduce the research/experiment without difficulty without further explanation. The student is able to justify the method and procedure followed. The student learns new skills quickly and needs little guidance. The student selects the most appropriate method of analysis and applies it independently. The student is able to apply more complex techniques with assistance. |

| Assessment criteria Oral presentation | | | |
| --- | --- | --- | --- |
| **Insufficient** | **Sufficient** | **Good** | **Excellent** |
| **Presentation skills** *Narration, time planning, use of audiovisual resources* | | | |
| The presentation is clearly too long or too short and difficult to follow. The audience does not feel addressed. Inadequate use of audiovisual resources. | Presentation meets time standard. Clear manner of presentation. Adequate use of audiovisual resources. | Presentation meets time standard. Enthusiastic and clear presentation style. Good use of audiovisual resources. Slides support the presentation. | The presentation met the time standard. Clear presentation with informative slides. Lively and enthusiastically presented. The audience is captivated by the presentation. |
